# What’s cooking? The effect of temperature on the immune response

**DOI:** 10.3389/fimmu.2025.1701016

**Published:** 2025-11-27

**Authors:** Julie M. Old, Brian Dixon

**Affiliations:** 1 School of Science, Hawkesbury, Western Sydney University, Penrith, NSW, Australia; 2 Department of Biology, University of Waterloo, Waterloo, ON, Canada

**Keywords:** endotherm, poikilotherm, mammal, teleost, immunity, virus, comparative immunology, infection

## Abstract

Animals have evolved to live in different environs, with some having developed specialised features to support survival in adverse conditions, whilst others adapt behaviourally. Temperature impacts the immune system differentially, with lower temperatures impacting the adaptive immune response, but not the innate response. A reduction in adaptive immune capacity at lower temperatures can be due to a failure to effectively assemble MHC and may be a result of modifications of the plasma membrane structure or its viscosities. Higher temperatures can be beneficial initially, but can be disruptive if excessive. Ensuring the effectiveness of the adaptive immune response through optimal temperature regulation or manipulation, where possible, may support immunological fitness, ultimately enhance the conservation and biodiversity of wildlife species, and improve welfare outcomes for production animals and ourselves.

## Introduction

Animals have adapted to suit the environments they live in. Ideally, they maintain body temperatures that favour their metabolic and physiological processes. Some vertebrates can metabolically maintain their internal body temperature to favour these processes (generally termed endotherms), whilst others utilise the environment to increase or decrease their body temperature (generally termed poikilotherms), and still others vary in their ability to maintain internal body temperature; thus, there is no clear demarcation between the terms endotherm and poikilotherm. Furthermore, whilst body temperature is species-specific, it can vary markedly even among members of the same species (1).

Extremes of temperature impact metabolic and physiological processes, and under these conditions, animals need to reduce their metabolism to conserve energy and survive. Some animals have developed extreme adaptations to endure these in harsh conditions, resorting to hypometabolic states. Animals living in environments with extreme ambient temperatures and that lack water or food may undergo dormancy (2, 3). When temperatures are high and available water is low, animals undergo aestivation (4), whereas in extremely low temperatures, animals undergo hibernation, and still others undergo bouts of arousal and torpor (5). However, it is not a one-size-fits-all situation.

Whilst much research has focused on the physiological processes impacted by varying body temperature, the impacts on the immune system and its response are lagging. However, raising an immunological response requires energy (6, 7) and can be dose- and time-dependent (8). This paper investigates what we know about the impact of temperature on the immune response, with a focus on a comparison of endothermic and poikilothermic animals.

## Endotherms *vs*. poikilotherms

Endothermy ensures that physiological functions including digestion, mobility, and decision-making can be carried out. Mammals and birds are “classic” endotherms (9, 10). Human body temperature is 37°C but varies between individuals (11). Monotremes and marsupials have a significantly lower body temperature compared to eutherians. Birds have higher body temperatures than mammals (10), and their thermoregulation is thought to occur through muscle hyperplasia, mostly by the pectoral muscles (12, 13).

Endothermy is, however, energetically costly because body temperatures are normally maintained above ambient temperatures. Mammals and birds are unique in that they have hair or fur, or feathers, to help with insulation. They can also decrease blood flow to the skin surface to reduce heat loss and conserve energy. Whilst one might expect mammals and birds living in extremely cold conditions to increase metabolic rates to ensure survival, it appears that this is not the case, rather some superficial tissues are maintained at the temperature of the environment (14). For example, reindeer (*Rangifer tarandus*), called caribou in North America, and gulls have temperature gradients that decrease along the tibial end of the legs. Both mass of the leg and fur decrease at this point of the leg in reindeer, whilst this portion of the gull leg is absent of insulating feathers (14). In some species, veins are situated close to arteries, enabling heat exchange from arteries to veins (counter-current heat exchange) rather than the heat being lost, ensuring heat conservation (14). The platypus (*Ornithorhynchus anatinus*), a monotreme, likely also uses a counter-current exchange system in its limbs, as well as its thick fur and a burrow that helps to maintain higher than ambient temperatures (15).

However, not all animals have fur or feathers to help with insulation. Marine mammals have a skin temperature that may be only one or two degrees warmer than the surrounding water (16), but they also utilise counter-current heat exchange (17). If cooling is required, blood flow is increased to the fins and flukes, and if heating is required, blood is kept closer to the animal’s core (17). Whales and dolphins rely solely on blubber as an insulator, which can be made up by as much as 93% lipid; however, seals and other marine mammals also have fur to enhance insulation (16). Seals and walruses (*Odobenus rosmarus*) can also “haul out” to warm. Some marine mammals, like terrestrial reindeer that undergo one of the largest migrations known (18), also move to warmer environments by migrating annually. Hence, some animals can exhibit behavioural adaptations to avoid the extreme cold, even if they are endotherms.

Endothermy in aquatic environments is problematic because of the high heat capacity of water; this is particularly problematic for fish where the gill lamellae and water come into close contact with the blood (19). Hence, many fish are regional endotherms and only able to increase the temperature of some specific tissues or organs (19). Regional endotherms include the tuna (Scombridae), lamnid sharks (Lamnidae), and billfishes (Istiophoridae and Xiphiidae) (19).

In tuna and some sharks, including the white shark (*Carcharodon carcharias*) and shortfin mako (*Isurus oxyrinchus*), they use a network of capillaries in their swimming muscles, the retia mirabilia, as a heat exchanger (20). Through counter-current exchange, the heat produced through muscle activity is transported by the blood. Through this metabolic process, sharks, for example, can maintain a body temperature above ambient water temperature. This process presumably provides an evolutionary advantage for these long-distance, migratory fish, allowing them to travel extensive distances and dive deep whilst maintaining body temperature, conserving energy, and avoiding thermal shock from changes in water temperature.

Regardless of animals being “endothermic” or poikilothermic, all species have an optimal temperature for physiological processes that are species-specific. Most fish and most reptiles and amphibians are “classically poikilotherms” and unable to metabolically heat their body, relying on the environment to heat or cool themselves. As observed by anyone that has ever owned aquarium fish, they will know some fish prefer colder water, whilst others prefer warmer water. The ideal body temperature required to maintain metabolic and physiological processes also varies between individuals. For example, a young rainbow trout (*Oncorhynchus mykiss*) that is growing compared to an older trout of reproductive age can vary in their temperature preference (21).

Poikilothermic animals are, however, restricted to the ambient temperature around them to perform physiological processes. In the case of fish, we could assume that “endothermic fish” would have an evolutionary advantage and be able to inhabit waters with more variable temperatures, as they are able to regulate their temperature. However, Harding et al. (22) found that this assumption is not the case. In a study comparing 16 endothermic fish (including 45 individuals), “endothermy” did not facilitate the use of broader thermal niches but rather only provided enhanced swimming performance (22). Hence, “endothermy”, in the case of fish, appears to not enhance habitat use but rather favour metabolic processes.

“True” poikilotherms have also adapted ways to survive harsh temperatures. Many fish species decrease food intake when temperatures decrease (23, 24). Cunner (*Tautogolabrus adspersus*) become dormant and downregulate metabolic enzyme activity (25, 26). Winter flounder (*Pseudopleuronectes americanus*) are freeze-tolerant due to the production of antifreeze proteins that bind to ice and inhibit ice crystal growth (27). Differences are also observed between amphibian and reptile species, with some migrating seasonally to find shelter, and others undergoing hibernation in their breeding grounds (28, 29), whilst wood frogs (*Rana sylvatica*) and some lizards and turtles can survive internal ice formation (30, 31).

Poikilotherms, like some endotherms, can also adapt behaviourally, for example, by migrating to warmer waters, or acclimatising when moderate changes in temperature occur, such as those associated with seasonal changes (reviewed in 23).

Thus, many animals have evolved the ability to metabolically produce heat to maintain their near-constant body temperature, whilst others adapt behaviourally to move to warmer or cooler environs. Subsequently, there are many different strategies that ultimately enable each species and each individual animal to perform their unique metabolic and physiological processes.

## Temperature affects disease occurrence regardless of the ability to metabolically produce heat

**Figure 1:**
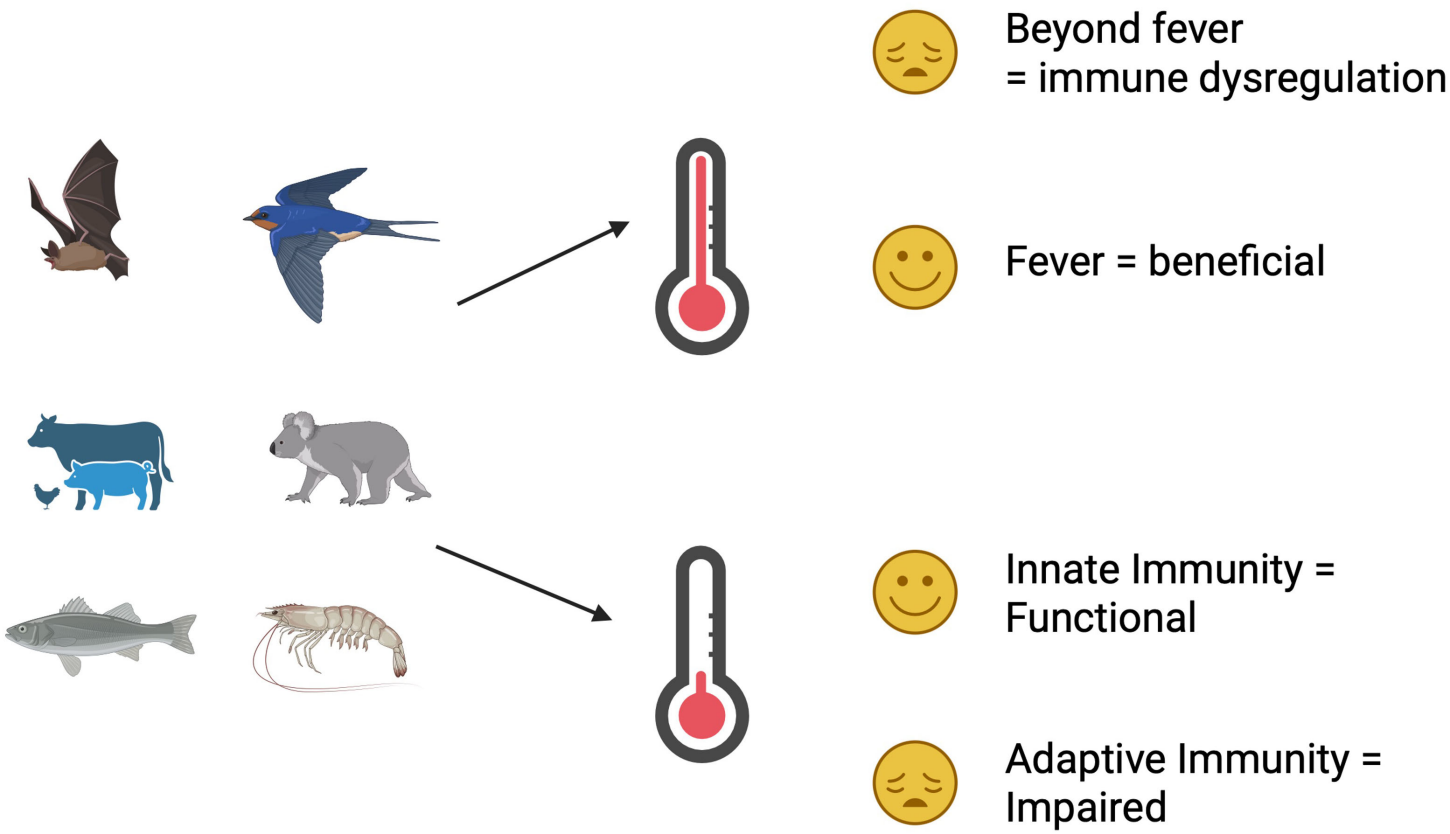
A summary (graphical representation) of the general themes in this paper. All animals regulate temperature, either internally or behaviourally, and are subject to extremes of their optimal range. Small temperature increases (fever) can be beneficial to host immune responses, but increases beyond that range are detrimental. Lower temperatures generally inhibit energetically expensive adaptive immune responses but generally leave innate immune mechanisms intact.

Whilst temperature has wide-ranging implications on physiological and metabolic processes, much less is known about the impacts on the immune response. As one might expect, when animals maintain their body temperature outside the temperature that maximises physiological and metabolic processes, it may induce stress, affording an advantage to pathogens, but also limits access to resources for pathogens and potentially impairs their ability to proliferate in the host. **Figure 1** provides a summary of the general themes of temperature and their effects on the immune response.

Angolan free-tailed bats (*Mops condylurus*) are suspected reservoirs for Ebola (32, 33). When inoculated with Ebola virus, high levels of viral replication and shedding occurs but no clinical disease (34). These bats undergo daily torpor in summer and winter, with mean body temperature ranging from 30 to 37°C at night (11), 38–41°C during flight, and being as low as 12°C during torpor (35). Han et al. (36) and Wang et al. (37) suggested that virus clearance in bat populations may be delayed due to reduced immune responses and virus replication rates during torpor, when body temperatures and metabolic rates are reduced.

Ebola virus replicates at 41°C (38). When compared to highly symptomatic host cells, including humans (39), cell cultures derived from the Angolan free-tailed bat exhibit lower Niemann-Pick C1 (NPC1) expression, a protein utilised by ebolavirus glycoproteins to enter cells (40–42). Bat cells remained viable, unaltered, and proliferated in suspension when incubated at 27°C, 37°C, and 42°C for up to 21 days, with cells cultured at 42°C slightly enlarged (43). When bat cells were cultured at 27°C, NPC1 expression levels were higher and viral replication rates decreased, whilst at 42°C, only a small reduction in viral replication was noted (43).

O’Shea et al. (44) suggested that some viruses may persist because of a resistance to the bat innate immune response, during daily periods of increased metabolic rates when flying, thus supporting any inherent costs of raising metabolism. More recently, Nemcova et al. (45) showed that greater mouse-eared bat (*Myotis myotis*) macrophages cultured at temperatures equivalent to hibernation, torpor, and euthermia (8°C, 17.5°C, and 37°C, respectively) were all able to proliferate, but were significantly reduced at 37°C. However, phagocytic activity and adherence to culture plates were reduced at lower temperatures, but were quickly restored when the temperature returned to 37°C, and even after freezing at −20°C and thawing, macrophages were viable and could proliferate (45).

Furthermore, Toshkova et al. (46) found that, at high temperatures, similar to those when flying, greater mouse-eared bat and common noctule (*Nyctalus noctula*) IgG demonstrated increased antigen binding strength and diversity for both pathogen-derived and auto-antigens. These differences are mediated by the variable regions of immunoglobulins (46). Bat IgG also exhibits low thermodynamic stability and high levels of polyreactivity, which differs from that of other mammals and birds (46).

Lu et al. (47) characterised the MHC I binding proteins (Ptal-N*01:01) derived from several bat-borne viruses and the bat MHC class I structures that they complex with. They found that bats had an unusual MHC class I presentation compared to other eutherians, including a three- or five-amino-acid insert in their peptide binding groove, and pairing of a negatively charged residue at position 59 and a positively charged residue at position 65 (47). These features may also provide insights into bat–virus interactions.

However, a reduction in viral replication at higher temperatures is not universal for all pathogens. In white-nosed disease caused by *Pseudogymnoascus destructans*, which infects bats, the fungus has an optimal growth at lower temperatures (8–14°C) such as those that bats reside at during hibernation. Subsequently, bats do not exhibit inflammatory responses to the fungus during hibernation as they would when not hibernating (48).

Clearly, some viruses are temperature-sensitive, meaning they do not replicate at the normal body temperature of the host and only replicate at lower temperatures (49). The occurrence of viruses only replicating at lower temperatures has even been observed in plants. Infected tobacco exhibits signs of disease at temperatures ranging from 20 to 30°C, but rarely showed signs of disease above 30°C and none at 37°C. Likewise, many common human respiratory viruses, such as rhinoviruses, replicate best at temperatures that are like the human respiratory airway (32 to 34°C) rather than mean body temperature (50, 51). Upper airway infections rarely reduce host mobility and mortality, induce limited immune responses, allowing recurrent infections, and trigger coughing and sneezing, which helps spread the infection and subsequently the virus (52). Avian influenza H5N1 virus outbreaks in birds have also been linked to reductions in temperature (53).

Lower temperatures also impact fish disease occurrence. Bennoit and Craig (54) found a delay in immune response and reduced oxygen consumption and, hence, metabolism at 22°C compared to 27.5°C in zebrafish (*Danio rerio*) when challenged with heat-killed *Vibrio anguillarum*. Levet et al. (8) similarly found that damselfish (*Pomacentrus amboinensis*) challenged with higher concentrations of lipopolysaccharide endotoxin resulted in higher oxygen uptake and, hence, increased metabolism. Viral haemorrhagic septicemia virus (VHSV) proliferates at lower temperatures when host fish have reduced immunological capacity (55), whilst the parasite *Flavobacterium psychrophilum*, which causes the aptly named coldwater disease, also impacts fish at temperatures less than 16°C but more severely at less than 10°C (56). However, at least in some fish species, like tilapia (*Oreochromis aureus*), acute cold stress is thought to result in immunosuppression, with increased plasma cortisol levels being correlated with decreased phagocytic activity of leucocytes (57). Acclimation responses to cold can be shifted in some species, such as perch (*Perca fluviatilis*), whereby phagocytic activity increases at lower temperatures (58).

Similarly, cold temperatures impact walleye (*Sander vitreus*) immune responses. Walleye are freshwater fish native to northern America. Age and longevity differ according to climate, with maturity occurring from 2 years in its southern range to 11 in its northern range, and longevity ranging from 5 years in the south to 30 years in their northern range (59). They are popular for sports fishing and as a food source, having been named the official provincial fish of Manitoba (60) and Saskatchewan (61), and the state fish of Minnesota and Dakota (62), and most interestingly in 2012 declared the “state warm water fish” of Vermont (63).

Walleye are affected by proliferative skin lesions called walleye dermal sarcoma (WDS) and walleye epidermal hyperplasia (WEHV). Both diseases are caused by retroviruses, WDS virus, and WEHV-1 and WEHV-2, in the *Epsilonretrovirus* genus (64). WDS lesions are randomly distributed mesenchymal neoplasms, up to 1 cm in diameter, originating from the superficial surface of scales (65–68). In contrast, WEHV lesions are translucent plaques of thickened epidermis of 2 to 50 mm in diameter and appear as localised hyperplasia of Malpighian cells (65–67). These diseases occur seasonally, with the highest incidence occurring from late fall/autumn until spring spawning, after which time they naturally regress (69), and are very rare in summer (69).

Transmission of the WDS retrovirus appears to occur via contact with infected water or directly during spawning (70). Experimental transmission studies have led to all infected fish at 6–8 weeks of age developing invasive tumours; however, in fish aged 12 weeks, only 2% developed tumours, and no fish developed tumours when infected at 52 weeks of age (71, 72), suggesting that higher temperatures likely inhibit retrovirus replication and also that maturation of the immune system enhances the ability to mount an immune response to the retrovirus. Additional experimental studies investigated the impact of rechallenge and found significantly lower numbers of tumours, compared to naïve fish (73). Subsequently, walleye only develop tumours after reaching sexual maturity and during their first participation in spawning, thereafter, remaining tumour-free (73). Regression of tumours leads to subsequent resistance.

Water temperature, rather than season, therefore affects WDS tumour development and regression (73, 74). When fish infected and held at 15°C, and after the development of tumours, were randomly assigned one of three temperature groups for a further five months, 3% of fish held at 10°C, 28% held at 15°C, and 32% held at 20°C exhibited tumour regression (73). Getchell et al. (74) suggested that all walleyes develop WDS over their lifetime; however, only half the walleye in a particular year-class will develop WEH during their lifetime (75).

Disease occurrence at cold temperatures is not limited to fish. Temperature also affects amphibian disease occurrence. *Batrachochytrium dendrobatidis* causes chytridiomycosis (76, 77) and has led to significant amphibian declines globally (78). However, the pathogen does not grow above 28°C (79) or in captive frog hosts exposed for short periods at temperatures between 27 and 37°C (80). Studies in free-ranging leopard frogs (*Rana yavapaiensis*) have similarly found low infection rates of chytrid (10%) in frogs living in water at higher temperatures (>30°C) compared to those living in water with lower temperatures (<15°C) (81). Hence, in this example, frogs living in higher temperatures are at lower risk of infection and enhanced survival. **Table 1** provides a summary of the effects of temperature on the immune response of vertebrates.

**Table 1:** Summary of immune responses to increased temperatures in vertebrates.

Immune feature	Response at elevated temperature	Immune type	Reference(s)
Neutrophil and monocyte motility/emigration	Increased	Innate	(82–85)
Phagocytosis and pinocytosis	Enhanced	Innate	(45, 57, 84)
Oxygen radical production	Increased	Innate	(84, 86)
Interferon production	Increased	Innate and adaptive	(84, 86, 87)
NK cell stimulation by interferon	Enhanced	Innate	(11, 46, 86)
Complement activation	Enhanced	Innate	(88, 89)
Fc receptor expression	Increased	Adaptive	(86)
T-helper cell activation and cytotoxicity	Increased	Adaptive	(90, 91)
T-suppressor cell activity	Reduced	Adaptive	(90)
Antibody production	Increased	Adaptive	(46, 84)
TNF-α production	Enhanced	Innate and Adaptive	(87, 91, 92)
T-cell proliferation and differentiation	Increased	Adaptive	(90, 91)
Host heat-shock proteins (HSPs)	Induced	Both	(87, 93–95)
MHC class I expression	Upregulated at optimal temperature	Adaptive	(96, 97)

## Effects of lowered body temperature on immune activity

Differences in the immune system of “hibernating” compared to non-hibernating mammals have been noted. Torpor in ground squirrels (*Callospermophilus lateralis*) reduces circulating leucocytes by approximately 90%; however, during the arousal phase, a dramatic increase in neutrophils and monocytes occurs, rising to those found in summer, whilst lymphocytes only increased to approximately 50% of those in summer (82). T-lymphocytes collected during torpor were approximately half that of those obtained from squirrels during arousal and summer (92). The increase in neutrophils occurs because of increased release of new neutrophils from the bone marrow and release of mature neutrophils from retention in tissues including the spleen and lungs (82). An extremely rapid return to large numbers of circulating lymphocytes during arousal can only be explained by the release of retained lymphocytes in the peripheral lymphoid tissues (82). Increases in the numbers of neutrophils and lymphocytes in the gut during torpor have also been noted by Inkovaara and Suomalainen (98) in hedgehogs.

Other immune responses have been noted in small mammalian hibernators. Complement activation and phagocytic capacity are reduced in golden-mantled ground squirrels during torpor (99). Skin allograft experiments in 13-lined ground squirrels (*Ictidomys tridecemlineatus*) suggest a lack of rejection during torpor but were rejected shortly after spring arousal (100). Furthermore, Con A-induced proliferation of artic ground squirrel (*Citellus undulatus pallus*) T cells exhibited half the proliferative capacity prior to hibernation and in torpor compared to those collected during arousal and summer (92). In addition, tumour necrosis factor-α (TNF-α) was higher in active and arousal periods compared to winter (92). Overall, it appears that the adaptive immune system is suppressed in hibernating mammals.

In contrast to true hibernators, which exhibit lowered metabolic rate and a dramatic drop in body temperature (101), brown bears (*Ursus arctos*) are also classified as hibernators, due to their 5- to 7-month-long Scandinavian torpid period (102). The American black bear (*Ursus americanus*) has also been defined as a hibernator as it has a 75% reduction in metabolic rate and a lowered body temperature, albeit not as low as that of small mammal hibernators (103).

Leucocyte counts of brown bears collected during hibernation were significantly lower than those in summer (83). Lower numbers of neutrophils and monocytes were detected, but there was no change in lymphocyte numbers, except for one bear that was excluded from the analysis (83). Hence, in bears, it appears that the innate immune system is suppressed during hibernation, and therefore, despite the similarities exhibited between many hibernating mammals, it appears that not all species undergo the same changes to their immune system. Apart from differences regarding body size, metabolic rate, and hibernation strategy, brown bears may rely on immune memory mechanisms and some pre-hibernation strategies or prioritise some immunological strategies over others during hibernation. Furthermore, as Flies and Consortium (104) suggest, this highlights the importance of understanding a range of species when studying the immune system and not relying solely on traditional laboratory models.

Tøien et al. (103) suggested that periodic arousal may have arisen due to the need to reactivate the immune system, and this may occur in small mammals as lymphocytes can be rapidly restored from the tissues to the blood. Frequent arousals where small mammals return to near-normal temperatures provide immune bursts, allowing accumulated pathogens to be cleared (82, 105). However, black bears do not exhibit spontaneous arousal to normal body temperature levels, and so, induction of an immune burst seems unlikely (83). Bears presumably exhibit an alternative strategy to clear pathogens during hibernation. Furthermore, during hibernation, bears, unlike other hibernating animals that are not able to maintain wound healing during hibernation due to hypothermia and reduced metabolism (105), are able to heal wounds (106).

A further study by Chow et al. (88) found during hibernation that American black bears increased expression of 34 serum proteins (<5%), the majority of which were related to immune system processes related to adaptive and innate immunity, complement activation, and the acute phase response, including α2-macroglobin, complement components, Ig μ heavy chain, and J chains. Subsequently, Chow et al. (88) suggested as the initial response to wound healing involves the innate response, including restoration of haemostatis and prevention of infections (107), that the changes observed in these immune-related proteins during hibernation may play an essential role. The ability to heal wounds and clear pathogens may be unique to bears and may be due to a reliance on local immune responses in the tissues, rather than a systemic immune response during prolonged hibernation; however, this requires further investigation as our knowledge of the underlying function of the immune system in hibernating bears is limited.

The impact of temperature on the immune response of other large cold-climate mammals is much less studied. Whales, seals, and reindeer, which utilise counter-current heat exchange, and animals that utilise regional endothermy use a combination of physiological adaptations to conserve energy and reduce heat loss to the environment. It seems plausible that their immune response may also be regional, and that perhaps like bears, they may focus their immune response in core areas. Alternatively, they may use these adaptations to enhance their ability to move to less pathogen-dense areas or move to areas to undertake behavioural fever. For ectothermic wood frogs, which freeze and rely solely on the environment to rewarm, a single immune burst may be triggered during the spring thaw to clear pathogens. As reviewed by Ferguson et al. (108), many ectotherms also maintain or upregulate innate defences whilst suppressing acquired defences, presumably decreasing costly immune defence and favouring less-specific defences that are not as resource heavy during cold temperatures. In Chinese alligator (*Alligator sinensis*), hibernating leads to changes in gut microflora, specifically an increase in bacterial taxa that degrade host mucin glycans, providing the host and microflora with energy and, when active, a Fusobacteria-rich microbiome, characteristic of a carnivorous diet (89). This change in gut microbiota associated with fasting has been observed in other species, as has hibernation (reviewed in 89). β-defensins were more highly expressed during hibernation in the alligator gut and, along with increasing levels of Toll-like receptor-2, occurred along the gut, with the highest levels found in the distal gut, where higher concentrations of pathogens were expected to occur (89).

Fasting and hibernation have also been shown to alter gut microflora in Syrian hamsters (*Mesocricetus auratus*) (109) and can subsequently impact the immune system (110, 111). Cytokine production by macrophages is reduced in pre-hibernating and hibernating artic ground squirrels (92). In hibernating ground squirrels, the numbers of intraepithelial lymphocytes, lamina propria leucocytes, and interferon γ (IFNγ), TNF-α, and interleukin-10 (IL-10) were higher than in summer, although intraepithelial cells were less mature, and B cells were found in higher numbers and T cells were found in lower numbers (111).

In teleosts, the endogenous antigen processing and presentation pathway (EAPP) is similar to that of mammals (112, 113) and recognised as an important antiviral pathway (114). A study using the rainbow trout monocyte/macrophage cell line RTS11 investigated the effect of poly IC and VHSV IVb for 14 days (96). The study demonstrated increased expression of MH class I heavy chain, β2-microglobin, and tapasin, with no change in expression of Erp57 or calreticulin when maintained at 14°C, and no changes in any expression levels when maintained at 2°C (96). However, a significant reduction in levels of β2-microglobin secretion was observed in both infected and non-infected cultures, suggesting that the viral infection reduced the expression of β2-microglobulin (and MH class I) at low temperatures, resulting in host susceptibility and subsequently disease (96).

A similar study using rainbow trout hypodermal fibroblast cells investigated the effect of poly IC and VHSV IVb on their EAPP (113). Cells incubated at lower temperatures did not vary in their transcription levels, but when exposed to poly IC and VHSV IVb, it induced upregulation of EAPP transcripts (113).

A further study using a walleye skin fibroblast cell line found that they did not vary in their transcript regulation response under varying temperatures (4°C, 14°C, 20°C, and 26°C) (115). However, when exposed to poly IC and VHSV IVb, the cells lost adherence, particularly at the lower temperatures (115). Furthermore, in the cells incubated at 20°C and 26°C, poly IC and VHSV IVb induced EAPP transcripts (*b2m*, *mhIa*, and *tapasin*), and at 4°C, the EAPP transcripts were delayed or completely impaired (115). Like mammals, lower temperatures appear to regulate the ability of walleye to fight viral infections and, thus, modulate the regulation of the EAPP, at least *in vitro* (115).

More recently, Wong-Benito et al. (97) investigated the protein dynamics of the EAPP in rainbow trout cells (monocyte–macrophage RTS11, gill epithelial RT-Gill-W1, and intestinal epithelial RTGut-GC) infected with VHSV IVb at 4°C, 14°C, and 20°C. MH class I and β2-microglobin were downregulated under suboptimal temperatures when measured 1, 4, and 9 days post-infection, and during trafficking of the MH class I/β2-microglobulin complex, some of the β2-microglobulin was released into the extracellular environment (97). Infection of RTGill and RTS-11 cells also resulted in significant upregulation of proteasome activity, but only at 14°C 9 days post-infection, potentially suggesting disruption of intracellular proteolysis at suboptimal temperatures as a result of increased peptide generation due to viral stress (97). Meanwhile, IFN-1 levels were significantly increased in RTGill and RTS11 at 14°C and RTS11 at 20°C from 4 days post-infection and in RTGut at 14°C but only at 4 days post-infection (97).

Other teleosts also exhibit immunosuppressive effects when in temperatures that are lower than their optimal range including olive flounder (*Paralichthys olivaceus*) to VHSV (116), zebrafish infected with spring viraemia carp virus (117), tilapia infected with *Streptococcus iniae* (118), and orange-spotted grouper (*Epinephelus coioides*) infected with *Vibrio alginolyticus* (119). Overall, in teleosts, there appears to be a reduction in the immune response at lower temperatures that is reversible when returned to warmer temperatures (120). The innate immune response of teleosts appears to remain functional at lower temperatures, compared to the adaptive immune response (120, 121), as observed in torpor of most hibernating mammals (82, 92).

Further studies are, however, needed to determine if the resultant impaired MH complex contributes to immunopathology or tolerance when teleosts are returned to their optimal temperature, but some studies have suggested that elevated temperatures impact the immune system, including the mucosal immune system, of Atlantic cod (*Gadus morhua* L.) and Atlantic salmon (*Salmo salar*). For example, Larsen et al. (122) found that despite the fact that cod infected with *Brucella pinnipedialis* had faster and more effective immune responses to the bacteria, their mortality rates were higher at 15°C when compared to 6°C. In addition, when cod were maintained long term at 17°C, they had a thinner epidermis, suggesting that their mucosal immune system may be compromised (123). Likewise, Thomsson et al. (124) found that higher temperatures led to a thinner epidermis, altered genes, and mucous properties in Atlantic salmon infested with sea lice (*Caligus rogercresseyi*). Also, Atlantic salmon alevins had better survival rates and mucosal immune defences against *Yersinia ruckeri* infections when raised between 4°C and 6°C, compared to those alevins raised at 8°C (125).

## Mechanisms of immune adaptation to thermal stress

Increasing body temperature (fever) is beneficial (11). It enhances both the innate and adaptive immune response (86), and can induce a thermal exclusion zone where pathogens are less adapted (126). Fever also promotes acute inflammatory responses in mammals that enables an initial response to pathogens (127).

Unsurprisingly, fever has been conserved throughout evolution (11). Mounting a higher temperature in endotherms to fight infections requires increased metabolic rates, and in humans, it has been estimated to be approximately 10% for every 1°C increase in temperature (128). Some mammals subsequently bask to assist with warming, presumably reducing the increased metabolic requirement. For example, kultarrs (*Antechinomys laniger*), red-tailed phascogales (*Phascogale calura*), and several other small marsupials bask to rewarm after torpor to conserve energy (129, 130). In contrast, poikilotherms must physically move to a warmer environment to increase their body temperature to limit pathogen growth and enhance immune defence. This process is termed behavioural fever.

Among reptiles, behavioural fever has been demonstrated in crocodilians, testudines, and squamates, but is not consistent across groups (131). For example, leatherback turtles (*Dermochelys coriacea*) use size, including insulating tissues, low metabolic rate, and basking on land to support lowering or raising body temperatures (132–134). They also use a counter-current heat exchange in their front and rear flippers (135). In contrast, gopher tortoises (*Gopherus polyphemus*) exhibit behavioural fever in response to LPS injection, regardless of season, exhibiting increased body temperature and heterophil:lymphocyte ratios, whilst plasma iron concentrations were reduced (131).

For fish, moving to warmer water has been shown to resolve bacterial infections faster than at colder temperatures (84). Even when goldfish that are uninfected move to warmer water (+2°C), their immune pathways are activated, indicating that warmer temperatures can enhance immune defences (84). It may also explain why some fish, such as young rainbow trout lacking a fully mature immune system, prefer higher temperatures than their adult conspecifics (21).

In contrast, abrupt changes in water temperature increase plasma cortisol levels in fish (136). A study in Atlantic cod exposed to 1°C temperature increases every 5 days from 10 to 19.1°C found that cortisol levels increased significantly at 16°C, whilst there was a slight increase in plasma glucose at 16°C and 18°C, with both cortisol and plasma glucose returning to control levels at higher temperatures (137). A similar pattern to that of cortisol occurred for β2-microglobin, MHC class I, and IgM light-chain gene expression, whilst IL-1β expression was significantly increased at 19°C and no change in IgM-H expression was observed throughout the experiment (137). Increasing temperature also had no effect on respiratory burst activity (137).

Heat shock proteins can protect cells from temperature stresses, performing roles as molecular chaperones and carrying out signal transduction (138). In fish, heat shock proteins are involved in both innate and T-cell-mediated immunity (139). Short-term heat shock may provide some advantage to fish. Guo et al. (87) found that an initial heat shock, followed by a second temperature challenge in rainbow trout led to a reduced stress response and upregulated heat shock protein expression (Hsp70 and hsc70). Both IL-1β and IL-6 transcripts were upregulated in spleen only, regardless of heat preconditioning, and no TNF-α upregulation was observed, whilst IFN-1 and MHC class I were downregulated in the gills at higher temperatures (87), suggesting that the temperature was too high for optimal function.

Furthermore, Atlantic cod exposed for 3 h to 18°C, and then returned to 10°C for 24 h, had liver, head kidney, and skeletal muscle samples collected prior to heat shock, at cessation of heat shock and 3, 12, and 24 h post cessation of heat shock and expression of upregulated and downregulated heat shock proteins assessed (93). HSP47-, GRP48-, and GRP94-like transcripts were significantly upregulated in all tissues (93). HSP90a and HSP70–1 were also significantly upregulated, but were upregulated more than 100-fold in liver samples (93). In contrast, Toll-like receptor 22 was significantly downregulated in the head kidney, and has been suggested as a component of heat-induced immunosuppression (93). Further research into the duration of acute stress and the point at which resolution of stress or chronic stress starts is needed. Many studies are only carried out over periods of hours, but longer studies could disentangle the effects of stress and lower temperature. Infection trials or immunostimulation experiments, similar to the behavioural fever trials currently being conducted, will be required to assess the correct amount of thermal change to benefit a particular species before the temperature effects start to have negative impacts.

More recently, McKenzie (140) investigated the effect of heat preconditioning prior to pathogen exposure, specifically *V. anguillarum*, in Chinook salmon (*Oncorhynchus tshawytscha*), and measured their immune response for 14 days post-exposure. No differences in the response to pathogen were observed between those preconditioned and those not preconditioned (140). Heat shock led to pro-inflammatory and anti-inflammatory responses due to increased IL-1-β, TNF-α, IL-8, and IL-10 3 days post-exposure, whilst hsp70 and hsp90 both increased expression in heat-shocked and pathogen-exposed fish (140). An earlier study investigating heat shock proteins also found that they were upregulated in Chinook salmon in response to haematopoietic necrosis virus (141). Overall, salmon benefited from heat shock and resulted in a preconditioning effect (140). Therefore, across vertebrate species, fever enhances adaptive immune responses and induction of heat shock proteins.

## Immunological processes involved

In humans and mice, fever is detected by pathogen-associated molecular patterns (PAMPs) including lipopolysaccharide, via pattern recognition receptors (PRRs) such as the Toll-like receptors (142). The inflammatory cytokines IL-1 and IL-6, produced by macrophages or dendritic cells, signal endothelial cells in the hypothalamus, inducing cyclooxygenase-2, increasing prostaglandin E_2_ synthesis, and increasing core body temperature (143).

O’Sullivan et al. (90) and Heintzman et al. (91) found that cultured murine CD8^+^ T cells, T-helper 1, T-helper 2, and induced regulatory T cells undergo increased rates of cell division at 39°C compared to 37°C, and CD4^+^ T cells exhibit an increased rate of differentiation into T-helper 2 cells (90, 91). CD8^+^ and T-helper 2 cells expressed higher levels of IFNγ, T-helper 17 cells expressed higher levels of IL-17, and induced regulatory T cells were less suppressive when cultured at higher temperatures (90, 91, 144). Furthermore, O’Sullivan et al. (90) and Heintzman et al. (91) found that CD8^+^ T cells and induced regulatory T cells had maximal oxygen consumption rates at 39°C, whilst T-helper 1 decreased and T-helper 17 remained unchanged. Heat shock at 40°C for 6 h also increases IFNγ production and oxygen consumption rates in γ δ T cells (94), whilst culturing monocyte-derived dendritic cells at 39°C led to a lasting decrease in oxygen consumption rates (145). Different T-cell populations thus exhibit different responses to increases in temperatures, perhaps suggesting why some differences may be exhibited between different species. Furthermore, following exposure at 40°C for 6 h, human γ δ T cells induced hsp70 expression, and after 3 days at 39°C, murine T-helper 1 cells increased hsp70 expression but was lost when temperatures returned to 37°C, with T-helper 17 and induced regulatory T cells exhibiting no changes in hsp70 expression (91, 94). As hsp70 supports protein folding and stability in response to stress (146), it may also contribute to decreased maximal oxygen consumption rate (142), via decreased oxidative phosphorylation (147). In mice, hsp90 increases adhesion and transmigration at higher temperatures, through binding and activation of α4 integrins in CD4^+^ and CD8^+^ T cells (148–150), thus suggesting improved immune cell activity (142).

In contrast, Ljunggren et al. (151) demonstrated that Reid’s murine lymphoma cells were unable to present antigen to cytotoxic T cells when maintained at 19°C to 33°C, being able to form an MHC class I molecule dimer with β2-microglobin but lacking a bound peptide. Rodrigues et al. (152) also found that when common carp (*Cyprinus carpio*) were maintained at lower temperatures, it led to decreased β2-microglobin and class I expression, and loss of cell surface expression when they were maintained at 6°C. However, when they were moved to higher temperatures, expression levels returned, albeit the group maintained at 6°C took longer to return to their original expression levels (152). MH class II α and β genes are downregulated at 2°C in rainbow trout (153). In contrast, Kales et al. (154) found no changes in β2-microglobin cellular or surface expression in rainbow trout or Atlantic salmon maintained at 2°C for 10 days. Furthermore, no changes in Ig expression were observed on common carp B cells (152), supporting the idea that cold temperatures specifically downregulate MHC genes required for effective adaptive immunity and likely result in disease, at least for some fish species.

Earlier, Bly et al. (155) found that channel catfish B-cell function kinetics, based on monoclonal antibody-induced membrane immunoglobulin capping, were affected by temperature, with lower energy activation occurring at lower temperature acclimation. Le Morvan et al. (136) subsequently reviewed the impacts of temperature on fish (e.g., channel catfish) and suggested that reduced temperatures suppressed T-helper cell responses, but not B-cell responses. Innate defences were also largely unaffected compared to adaptive defences (136), which is similar to that observed in small hibernating mammals. Hence, whilst there are differences in how endotherms and poikilotherms, and those in-between, regulate their body temperature, and subsequently when for their body temperatures are below the temperature ideal metabolic and physiological processes to occur, they appear to favour for innate immune defence mechanisms over more energy-rich adaptive immune defences.

It has been suggested that reduced environmental temperatures may lead to the modification of the common carp plasma membrane structure and subsequently affect its function (136, 152). This lack of function is similar to the findings of Ljunggren et al. (151) for the mouse, whereby MHC class I molecules can assemble at the optimal temperature for metabolic and physiological processes, but are unstable when peptides are lacking at the cell surface. However, Bly et al. (155) have also suggested that temperature impacted channel catfish B-cell immune function, specifically antibody-induced membrane immunoglobulin capping, through changes in plasma membrane viscosities. Hence, in mammals and teleosts, reduced temperature appears to disrupt antigen presentation, whilst in teleosts, it also impairs B-cell receptor dynamics, specifically B-cell activation and antibody production.

## Impacts of climate change and temperature variability on the immune system of vertebrates

Climate change is upon us with increases in extreme and unpredictable weather and mean temperatures (156). These changes are expected to impact biodiversity, food production (157), and human welfare. Animals adapted to live in cooler climates will need to move to avoid increased temperatures. Terrestrial species will need to move to higher altitudes or closer to the poles (158). Marine and freshwater fish distribution will also change, and their habitats will reduce (158). Impacts on fisheries will occur, and subsequently their sustainability (159). Animals unable to move or adapt will be placed under increased stress due to reduced food resources (160), resulting from disruptions to plant phenology (161) and, subsequently, plant communities, due to environmental disturbance.

In Australia, for example, climate change has led to increased fire risk, with the 2019–2020 fires estimated to have killed 2.8 billion terrestrial vertebrates and unknown numbers of marine and aquatic vertebrates and invertebrates (162). Extreme changes due to climate will lead to reduced plant food resources and decreased habitat and shelter, placing already at-risk species under additional stress, and at further risk from disease.

The immune system of wildlife species, particularly those living in extreme temperatures, are rarely studied to the same level as those we utilise as production or companion animals, because they are more difficult to maintain in captivity, and are often threatened species. For example, the Australian marsupial mountain pygmy possum (*Burramys parvus*), listed as critically endangered (163), has not been studied at all immunologically, despite some studies focusing on its ecology, diet, and thermal physiology (5, 164–166, 178).

Likewise, reindeer are cold climate specialists. They are critically important traditionally and culturally to the many Indigenous peoples of the arctic tundra and boreal forests (18, 167), and essential to its ecological health (168, 169). However, despite their ecological importance, reindeer are listed as Vulnerable on the IUCN Red list, having suffered a 40% decline in population numbers over the last few decades (18). The reason for the decline varies between subspecies; however, human activities associated with farming, mining, forestry, and hunting disturb migratory patterns, and additional threats, such as climate change, are placing further threats on the species. Trondrud et al. (170) recently described reduced reindeer activity, increased body temperature, and reduced heart rates, ultimately leading to reduced foraging time and decreased body mass in summer, because of an extreme heatwave in Finland. Heat waves, because of climate change, will impact cold climate specialists, such as reindeer, through reduced foraging times, reduced body mass, and subsequently reduced winter energy reserves, ultimately impacting their ability to successfully mount immune responses. Furthermore, when compared to many other production animal species, studies on free-ranging reindeer relate to specific pathogens and parasites and limited immunological studies (171, 172).

The equally iconic polar bear (*Ursus maritimus*) has one study investigating the impact of climate change on the species, and subsequently reduced distribution is anticipated due to decreased sea ice and, subsequently, resources, ultimately impacting the immune system (173). For marine mammals, loss of ice will increase land-based runoff, freshwater discharge, and toxic algal blooms, all of which have shown to increase contaminants with immunosuppressive effects (174) and subsequently enhancing their vulnerability to pathogens and parasites.

Some species will benefit from increased temperatures and rainfall including terrestrial ectoparasites. Tick activity has been suggested to increase as a result of climate change due to milder winters and will lead to increased diseases (175). Increases in disease occurrence among vertebrates will accelerate the loss of species and lead to biodiversity loss.

Impacts have already been observed in coral immune systems (176), whilst these are invertebrates, it seems likely that similar immune challenges will occur in vertebrates resulting from the impacts of climate change. For example, the complement pathway has been shown to respond to heat stress, albeit differently for different species, and is likely a critical pathway (176). Future studies therefore need to enhance our understanding of what the impacts of higher temperatures will have in the immune system of more diverse vertebrate and invertebrate species, and what it means for biodiversity.

## Conclusions and future directions

Clearly, we have much to investigate to better understand the role of temperature on the immune response of vertebrates, whether they be endotherms or poikilotherms, or somewhere in-between. In most cases, lower temperatures reduce the ability of the adaptive immune response to react effectively and provide further opportunities for viruses and other pathogens to replicate, but the innate immune response appears largely unaffected. However, it is not a one-size-fits-all scenario, and as expected, pathogens have evolved and adapted to avoid their host’s immune defences.

Nevertheless, it appears that manipulating the body temperature of some vertebrate species could, in the short term, enhance their immunological response to certain pathogens, thus enhancing immunological fitness. For example, increasing host temperature has shown some success in treating some frogs with chytridiomycosis in captivity (80). There are many studies on manipulating ambient water temperature for the improvement of disease resistance in aquacultured species, but clear effects of the application of this method are few. One recent clear example was the observation that vaccinating salmon at higher water temperatures radically increased efficacy and survival to disease challenge (177). This vaccine is now recommended for use with higher water temperatures. Behavioural fever (84) also offers promise for application of temperature manipulation for benefits in the real world, but all of these techniques must be balanced with the cost of heating water by producers. Whilst these treatments are likely beneficial in a captive situation, for species with a known maximum tolerable temperature, it may be difficult to determine a desirable temperature for all species, without inducing stress. In the longer term, increased temperatures may lead to increased energy expenditure and resource stress because of a higher metabolic rate and food and water requirements, as well as conflicts with phenology and changes to their physiology and behaviour, ultimately leading to variations in populations and potentially selection of a thermally tolerant pathogen. Infection trials or immunostimulation experiments, similar to the behavioural fever experiments already undertaken, will be required to assess the correct thermal change to benefit a particular species prior to any negative or adverse impacts occurring.

Maintaining bats susceptible to white-nosed disease in temperatures above those normally associated with hibernation may enhance inflammatory responses, reduce viral loads, and subsequently reduce incidence of the disease. Likewise, limiting temperature reduction in Angolan free-tailed bats may support Ebola viral clearance, ultimately reducing viral shedding and subsequently viral transmission to other species, including ourselves. Bats may even benefit from regular arousals to enhance the adaptive immune response, and help to maintain large numbers of circulating lymphocytes, as observed in small hibernating mammals (82), to reduce viral loads.

Outbreaks of avian influenza may be reduced by maintaining poultry and waterfowl at temperatures to ensure optimal metabolic activity. Temperature could be used to maximise the potential adaptive immune response through enhanced T-cell activity, ultimately reducing viral replication, transmission, and mortalities. Increasing the housing temperature may also result in increased temperature in the respiratory tract and reduced viral replication due to a suboptimal replication temperature. Furthermore, ensuring fish in production and vaccination systems are maintained at the optimal temperature for metabolic and physiological responses will likely support their adaptive immune response, reduce viral loads, and enhance the growth of individuals through improved health and welfare. Environmental temperature regulation could also reduce disease mitigation costs and disease spillovers into the environment. The practical challenges in manipulating the temperature of both domesticated and especially free-ranging animals are still large. Heating water for fish in tanks is possible but may be prohibitively expensive, especially in colder climates like Canada or Norway, for example.

In most cases, a little short-term cooking to enhance the immune system may be beneficial in helping to reduce disease incidence. Maintaining higher temperatures in host species appears to enhance adaptive immune responses necessary for specific immune defence and may benefit the conservation of wildlife species under threat from disease, as well as providing improved animal welfare outcomes for species in production systems and ultimately ourselves.
